# Spatial clustering and risk factors for late HIV diagnosis in the Yinzhou, China: a population-based retrospective cohort study

**DOI:** 10.3389/fpubh.2026.1888278

**Published:** 2026-10-27

**Authors:** Yijin Chen, Wei Yu, Bin Wang, Bingyang Liu, Haibo Jiang, Yi Gu, Lv Lin, Deguang Gong

**Affiliations:** 1 Ningbo Institute of Innovation for Combined Medicine and Engineering, Ningbo Medical Center Li Huili Hospital, Ningbo, China; 2 Department of Tuberculosis and HIV/AIDS Prevention and Control, Yinzhou District Center for Disease Control and Prevention, Ningbo, China; 3 Department of Tuberculosis and HIV/AIDS Prevention and Control, Ningbo Municipal Center for Disease Control and Prevention, Ningbo, China; 4 Baizhang Subdistrict Community Health Service Center, Yinzhou District, Ningbo, China

**Keywords:** health inequalities, late HIV diagnosis, older adults living with HIV, risk factors, spatial analysis

## Abstract

**Background:**

Late HIV diagnosis is a critical challenge for controlling the transmission; if not addressed, it could inevitably slow or reverse current progress toward the UNAIDS 95-95-95 targets by 2030.

**Methods:**

We conduct a retrospective study of a full registry of 1404 HIV cases in Yinzhou District (2001–2025), China. Adjusted relative ratio (aRR), posterior relative ratio (RR), Global Moran's I index, and adjusted hazard ratios (aHR) of late HIV diagnosis were calculated using Poisson's regression analysis, subgroup analysis, spatial autocorrelation analysis, and Cox regression, respectively.

**Results:**

The proportion of late HIV diagnosis was 76.4% in Yinzhou. Older age (aRR = 1.003, 95%CI: 1.001–1.006) at HIV diagnosis was significantly associated with a higher risk of late diagnosis. Patients who tested voluntarily (aRR = 0.918, 95%CI: 0.849–0.993) and living in the northwest (aRR = 0.933, 95%CI: 0.876–0.994) were associated with a lower risk. The northwest was a cold spot, and the central and southeast areas were hotspots for late HIV diagnosis (global Moran's *I* = 0.65, *p* < 0.001). Patients aged 60 and over and tested non-voluntarily was identified as a priority subgroup (posterior RR = 1.097, 95%PI: 1.013–1.174). Late presenters had a higher hazard of death than non-late presenters (aHR = 3.03, 95%CI: 1.04–8.84).

**Conclusion:**

We identified a priority subgroup at high absolute risk and a hotspot for late HIV diagnosis in Yinzhou. This finding provides evidence for developing targeted intervention strategies.

## 1 Introduction

Despite early diagnosis and treatment can significantly reduce the transmission of the human immunodeficiency virus (HIV) and mortality, there were still approximately 5.3 million people living with HIV (PLWH) remain unaware of their HIV status globally (1, 2). Late diagnosis of HIV, defined as CD4 count < 350/μl or with an acquired immunodeficiency syndrome (AIDS)-defining event regardless of the CD4 cell count, has become a growing concern in recent years as it mitigates the effect of early diagnosis, increases the risk of transmission, and worsens the PLWH's health outcomes (3). Notably, the burden of late diagnosis is prevalent worldwide; across 23 counties in the European Union and Economic Area, 49.6% of new HIV cases diagnosed between 2022 and 2024 were classified as late diagnosis (4). In Australia, the proportion of late HIV diagnosis cases in 2024 was 38.4% (5). In Florida, USA, approximately 41% of cases diagnosed between 2015 and 2021 were late diagnosis (6). Recent studies in China indicate even higher rates: the figure for Wuhan in 2023 was 67.89% (7), while in Hunan Province, it was 66.6% between 2018 and 2021 (8).

To reduce late diagnosis, expanding HIV testing coverage seems the most straightforward strategy, but where to expand is crucial. The impact of expanding testing coverage in hospitals has remained inconclusive. In Southwestern China, a concentrated HIV epidemic area, providing opt-out HIV testing for every outpatient significantly increases HIV positive cases (9). However, studies from low-epidemic areas showed no significant increase in the number of new diagnoses and were not cost-effective, as this would miss asymptomatic HIV infected individuals who don't seek medical care (10, 11).

Disparities in social and structural contexts shape the different testing behaviors of PLWH. For example, studies from Brazil and Southwestern China found that men who have sex with men (MSM) were at lower risk for late HIV diagnosis (12, 13), whereas a study from central China reported a higher risk among MSM (8), possibly because different regions have different key populations and corresponding campaign preferences, influencing risk perception and testing behavior among MSM (14). Furthermore, studies have found geographical heterogeneity in late HIV diagnosis, suggesting that environmental or structural factors may influence individual behavior (15). Therefore, exploring risk factors for late HIV diagnosis and understanding barriers to HIV testing in the local area are crucial for identifying undiagnosed PLWH populations and designing targeted, evidence-based strategies.

In this study, we will examine the proportion, risk factors, outcomes, and spatial distribution of late HIV diagnosis in Yinzhou District, Ningbo. Ningbo, an economically developed coastal city in southeastern China, had an HIV/AIDS rate of 8.38 per 100,000 in 2021, which is lower than the national average of 10.56 (16). However, research on HIV late diagnosis in Ningbo remains underexplored. A study in 2018 discovered the proportion of late HIV diagnosis in a district of Ningbo was 36.7%, and the definition they used to classify late presenters was CD4 less than 200/μl (17). The cutoff was stricter, which may undercount cases. Therefore, this study aims to provide up-to-date evidence to inform local policymakers and stakeholders in developing targeted policies for the timely identification of PLWH and optimizing resource allocation for HIV testing to prevent transmission and adverse outcomes.

## 2 Method

### 2.1 Study setting

As of 2024, Yinzhou District had an estimated 1.7 million residents, and per capita disposable income was 84531 RMB (18). There were three HIV/AIDS voluntary counseling and testing clinics, one HIV/AIDS designated hospital, and one HIV post-exposure prophylaxis clinic in Yinzhou, all located in the northwestern part of Yinzhou (19). The northeast areas are entirely urban, and the rest of the district exhibits a balanced mix of urban-rural settlements.

### 2.2 Study design

This study used two independent datasets extracted from the China National HIV/AIDS Comprehensive Response Information Management System: a full registry of 1505 HIV-positive cases from Yinzhou District, Ningbo (2001–2025), and a randomly selected 400 HIV-positive cases from Ningbo (2007–2025). Specifically, the 400 cases from Ningbo and the 1,505 cases from Yinzhou do not overlap. Hospitals and disease control centers across the country will report HIV-positive cases and follow-up information to the system in real time, including socio-demographic and behavioral information, laboratory results, antiviral treatment plans, and outcomes. The main analysis will be conducted on the Yinzhou dataset, and the Ningbo dataset will serve as an external validation dataset.

The outcome is late HIV diagnosis in multivariate analysis, subgroup analysis, and spatial analysis. Death is the outcome in survival analysis. Based on the definition, CD4 less than 350/μl or any AIDS-related symptoms presentation were grouped into late HIV diagnosis group.

The variables include: sex at birth, age at diagnosis, patient's registered address, patient's current address, clinic address, occupation, educational level, marriage status, testing behavior, year of diagnosis, sexual behavior, STIs history, AIDS-related events, HIV WHO stage, initial viral load, follow-up status, and initial CD4 count. All patient information was de-identified before analysis.

101 cases and 76 cases without a record of CD4 count were excluded from the Yinzhou dataset and the Ningbo dataset. The Ningbo Municipal and the Yinzhou District Center for Disease Control and Prevention ethical committees approved this study. The results of this research are reported in accordance with the STROBE statements (20).

### 2.3 Statistical analysis

R Studio version 2025.05.1 + 513 and Python version 3.10.18 were used for data cleaning and analysis. Before modeling, continuous variables with extreme values were winsorized at the 1st and 99th percentiles. Missing values were imputed using the multiple imputation by chained equations (MICE) method, primarily addressing missing rates of 51.85% for the number of non-marital heterosexual partners and 38.68% for same-sex partners. We compared variable distributions before and after imputation using descriptive statistics and standardized mean differences (SMDs), and conducted sensitivity analyses to assess the robustness of the imputation results, detailed in the Appendix R4.3.

The geographic distance between the patient's home address and the treatment clinic was obtained via the Baidu Maps APIs.

For ordinal variables such as educational level, a likelihood ratio test was performed prior to multivariable modeling to test for departure from linearity, and no significant deviation was observed (*p* = 0.937).

We then applied Poisson regression with a robust standard error variance to estimate relative risks (RRs), rather than odds ratios, given the common outcome and to facilitate proper interpretation. Each variable was first examined in a univariate model, and those with a *p*-value < 0.10 were included in the multivariable model. Both crude and adjusted RRs were reported to compare the magnitude of associations before and after adjustment for covariates. External validation was performed in an independent cohort, with baseline comparability assessed using standardized mean differences (SMD). We used CD4 count less than 200/μl as a definition of late HIV diagnosis to conduct a sensitive analysis to assess risk factors. It demonstrated that our findings are robust under this stricter definition. The direction of the associations remains consistent with the primary model, and variables age at diagnosis, student status, Northwest region, and voluntary testing remain statistically significant predictors.

### 2.4 Survival analysis

The survival probability and hazard of death associated with late diagnosis were assessed with Kaplan–Meier survival curves and Cox proportional hazard models. The outcome was all-cause mortality. Participants were censored at the date of last follow-up or study end. The exposure of interest was late diagnosis. We first fitted a crude Cox proportional hazards model including only the main exposure variable to estimate its unadjusted association with the outcome. The model was then adjusted for sex at birth, educational level, marriage status, testing behavior, and occupation, which were identified as potential confounders through domain knowledge and confounding analysis (Supplementary Table S1). Proportional hazards assumptions were tested with Schoenfeld residuals. Violations were detected for age and marital status. Subsequently, analyses were also done according to marital status until assumptions were satisfied (Supplementary Table S2). As a sensitivity analysis, models were fitted using attained age and diagnosis time as the underlying time scale, respectively. The change-in-estimate after adjustment showed that models based on age were more stable compared with diagnosis time as the time scale, thus age-based models are presented as the primary analysis.

### 2.5 Subgroup analysis

The study population was divided into multiple strata according to age group and testing behavior. We performed stratified grid methods with empirical Bayes (EB) shrinkage to stabilize effect estimates in smaller strata (21). For each stratum, we computed the raw event rate with 95% Wilson confidence intervals and the shrinkage-adjusted rate (posterior mean) with 95% posterior intervals (PI) obtained by Monte Carlo sampling from the posterior beta distribution. Relative risks of each stratum were assessed with the entire sample as the prespecified reference stratum.

### 2.6 Spatial distribution and autocorrelation analysis

We applied the EB smoothing method to mitigate differences in resident size and thus stabilize estimates of observed rates. The geographic data of Yinzhou District was downloaded from OpenStreetMap, available under the Open Database License. We created a choropleth and bubble map to visualize the proportion of late HIV diagnosis and the number of HIV cases across 25 sub-districts. Next, we performed Global Moran's statistics to calculate the spatial clustering of the proportion of late HIV diagnosis and identified Local Moran's I index and significance for each street in clusters in Yinzhou District. When the value of Moran's I is not equal to 0, it means there is spatial clustering.

## 3 Results

There were 1404 patients diagnosed with HIV/AIDS in Yinzhou District, Ningbo, between April 2001 and June 2025 analyzed, including 1215 (86.5%) males and 189 (13.5%) females, with a median age at diagnosis was 34 years, as summarized in Table 1.

**Table 1:** Characteristics of 1404 HIV infected patients in Yinzhou district of Ningbo, grouped by late diagnosis and non-late diagnosis of HIV.

Characteristics	Overall *N* = 1,404	LD *n* = 1,072 (76.4%)	Non-LD *n* = 332 (23.6%)	LD within category, *n/N* (%)
Age at diagnosis, Median [Q1, Q3]	34.0 [26.0, 47.0]	35.0 [27.0, 49.0]	30.0 [24.0, 41.0]	
Driving distance to clinic (km), Median [Q1, Q3]	6.1 [3.5, 9.7]	6.1 [3.5, 9.8]	6.2 [3.5, 9.3]	
No. of non-marital heterosexual partners, Median [Q1, Q3]	2.0 [2.0, 4.0]	2.0 [1.0, 3.8]	2.0 [2.0, 4.0]	
No. of same sex partners, Median [Q1, Q3]	5.0 [3.0, 10.0]	5.0 [3.0, 10.0]	5.0 [3.0, 8.0]	
Sex at birth, *n* (%)				
Female	189 (13.5%)	148 (13.8%)	41 (12.3%)	148 (78.3%)
Male	1215 (86.5%)	924 (86.2%)	291 (87.7%)	924 (76.0%)
Marriage status, *n* (%)				
Single	664 (47.3%)	471 (43.9%)	193 (58.1%)	471 (70.9%)
Married/partnered	528 (37.6%)	422 (39.4%)	106 (31.9%)	422 (79.9%)
Divorced/widowed	210 (15%)	178 (16.6%)	32 (9.6%)	178 (84.8%)
Unknown	2 (0.1%)	1 (0.1%)	1 (0.3%)	1 (50.0%)
Education, *n* (%)				
Primary or below	223 (15.9%)	190 (17.7%)	33 (9.9%)	190 (85.2%)
Lower secondary education	385 (27.4%)	302 (28.2%)	83 (25.0%)	302 (78.4%)
Upper secondary education	270 (19.2%)	206 (19.2%)	64 (19.3%)	206 (76.3%)
Tertiary education	526 (37.5%)	374 (34.9%)	152 (45.8%)	374 (71.1%)
Occupation, *n* (%)				
Services and sales workers	553 (39.4%)	408 (38.1%)	145 (43.7%)	408 (73.8%)
Government employees and professionals	88 (6.3%)	69 (6.4%)	19 (5.7%)	69 (78.4%)
Manual and agricultural workers	287 (20.4%)	237 (22.1%)	50 (15.1%)	237 (82.6%)
Fishery workers	6 (0.4%)	4 (0.4%)	2 (0.6%)	4 (66.7%)
Not in the labor force	330 (23.5%)	256 (23.9%)	74 (22.3%)	256 (77.6%)
Student	56 (4.0%)	33 (3.1%)	23 (6.9%)	33 (58.9%)
Others	76 (5.4%)	58 (5.4%)	18 (5.4%)	58 (76.3%)
Orientation, *n* (%)				
Northeast	68 (4.8%)	55 (5.1%)	13 (3.9%)	55 (80.9%)
Southeast	40 (2.8%)	32 (3.0%)	8 (2.4%)	32 (80.0%)
Central	95 (6.8%)	75 (7.0%)	20 (6.0%)	75 (78.9%)
Northwest	1060 (75.5%)	798 (74.4%)	262 (78.9%)	798 (75.3%)
Southwest	141 (10.0%)	112 (10.4%)	29 (8.7%)	112 (79.4%)
Partner's HIV status, *n* (%)				
Negative	167 (11.9%)	131 (12.2%)	36 (10.8%)	131 (78.4%)
Positive	121 (8.6%)	100 (9.3%)	21 (6.3%)	100 (82.6%)
Not tested/Unknown	1116 (79.5%)	841 (78.5%)	275 (82.8%)	841 (75.4%)
STIs history, *n* (%)				
Yes	262 (18.7%)	196 (18.3%)	66 (19.9%)	196 (74.8%)
No	1068 (76.1%)	824 (76.9%)	244 (73.5%)	824 (77.2%)
Unknown	71 (5.1%)	49 (4.6%)	22 (6.6%)	49 (69.0%)
Testing behavior, *n* (%)				
Voluntary testing	642 (45.7%)	459 (42.8%)	183 (55.1%)	459 (71.5%)
Compulsory testing	468 (33.3%)	376 (35.1%)	92 (27.7%)	376 (80.3%)
Opportunistic testing	294 (20.9%)	237 (22.1%)	57 (17.2%)	237 (80.6%)
Similar symptoms in partner(s), *n* (%)				
No	1337 (95.2%)	1015 (94.7%)	322 (97.0%)	1015 (75.9%)
Yes	67 (4.8%)	57 (5.3%)	10 (3.0%)	57 (85.1%)
Sexual behavior^a^, *n* (%)				
Sexual contact with an HIV-positive partner	85 (6.1%)	67 (6.2%)	18 (5.4%)	67 (78.8%)
Non-marital commercial sexual contact	265 (18.9%)	204 (19.0%)	61 (18.4%)	204 (77.0%)
Non-marital sexual contact	279 (19.9%)	220 (20.5%)	59 (17.8%)	220 (78.9%)
MSM contact	673 (47.9%)	490 (45.7%)	183 (55.1%)	490 (72.8%)
Others	17 (1.2%)	14 (1.3%)	3 (0.9%)	14 (82.4%)
Possible transmission route, *n* (%)				
Heterosexual transmission	722 (51.4%)	576 (53.7%)	146 (44.0%)	576 (79.8%)
Same-sex transmission^b^	671 (47.8%)	488 (45.5%)	183 (55.1%)	488 (72.7%)
Others	11 (0.8%)	8 (0.7%)	3 (0.9%)	8 (72.7%)

^*a*^Sexual behavior was a multiple-choices question, and those who responded “Yes” were included in the table.

^*b*^Only male responded to this information.

LD, late diagnosis; STIs, sexually transmitted infections.

1072 (76.4%) patients were diagnosed late, and 332 (23.6%) were diagnosed not late. Most characteristics showed comparable proportions across the two groups. The majority were male in both groups (86.2% in the LD group and 87.7% in the non-LD group) and the proportion of late diagnosis was 76.0% among males and 78.3% among females. Additional category-specific proportions are presented in Table 1. The majority were lived in the northwestern part of Yinzhou (74.4 and 78.9%). Services and sales were the most common occupations in both groups (38.1 and 43.7%). For the behavior characteristics, patients in both groups were mostly diagnosed via voluntary testing (42.8 and 55.1%). Educational background differs across groups, with secondary education predominating among late presenters (47.4%), and tertiary education predominating among non-late presenters (45.8%).

The external validation dataset included 324 HIV/AIDS patients, of whom 249 (76.9%) were diagnosed late and 256 (79.0%) were male, as shown in Supplementary Table S3. Several different characteristics were observed between the two datasets, such as the median age at diagnosis was older in Ningbo than in the Yinzhou District (47 vs. 34 years), the most common occupation is manual and agricultural workers, and 50% cases were diagnosed compulsively in Ningbo.

Supplementary Figure S1 shows the changes in the proportion of late HIV diagnosis and the number of new HIV cases from 2002 to 2024. Over the past decade, the proportion of late diagnosis has fluctuated without a clear monotonic trend, but has generally increased from 64.9 to 74.7%. The number of new HIV cases has also fluctuated, decreasing overall from 117 to 85.

### 3.1 Factors associated with late HIV diagnosis

The result of univariate analysis identified age, marriage status, educational level, as a service and sales worker, as a manual and agricultural worker, as a student, living in the northwest area, a HIV-positive partner, testing behavior, partner(s) had no similar symptoms, and had MSM contact were significantly associated with HIV late diagnosis. These factors were further included in multivariate analysis, as shown in Table 2. Age was considered as a continuous variable, older age at HIV diagnosis was significantly associated with a higher risk of late diagnosis. Specifically, each year increase in age was associated with a 0.3% higher adjusted risk of late diagnosis (aRR = 1.003, 95%CI: 1.001–1.006). Patients living in the northwest areas had a 6.7% lower adjusted risk of late diagnosis compared with those living in the northeast areas (aRR = 0.933, 95%CI: 0.876–0.994). For the testing behavior, patients who tested voluntarily had an 8.2% lower adjusted risk of late diagnosis compared with those who were diagnosed opportunistically (aRR = 0.918, 95%CI: 0.849–0.993). Older age was also found in the external validation dataset (aRR = 1.007, 95%CI: 1.002–1.012) in Supplementary Table S4. Voluntarily testing behavior was not statistically significantly related to late HIV diagnosis in Ningbo, likely due to differences in testing behavior in the district and the city. Orientation is an intra-district geographic categorization variable and was therefore not analyzed in the external dataset.

**Table 2:** Univariate analysis and multivariate analysis of factors associated with late HIV diagnosis in 1404 HIV infected patients in the Yinzhou district.

Variables	Crude RR	95% CI	*p*-value	Adjusted RR	95% CI	*p*-value
Age at diagnosis	1.005	1.004–1.007	< 0.001	1.003	1.001–1.006	**0.032**
Driving distance to clinic (km)	1.001	0.996–1.005	0.823	–	–	–
Sex at birth						
Female	Reference					
Male	0.971	0.895–1.054	0.481	–	–	–
Marriage status						
Single	Reference					
Married or partnered	1.077	1.017–1.141	0.012	1.012	0.927–1.104	0.793
Divorced or widowed	1.132	1.060–1.209	< 0.001	1.084	0.988–1.190	0.088
**Educational level**	0.945	0.922–0.969	< 0.001	0.989	0.958–1.022	0.517
Occupation						
Services and sales workers	0.946	0.889–1.005	0.073	0.994	0.925–1.068	0.869
Government employees and professionals	Reference					
Manual or Agricultural workers	1.105	1.037–1.177	0.002	1.040	0.968–1.117	0.288
Fishery workers	0.873	0.495–1.538	0.637	–	–	–
Not in the labor force	1.023	0.957–1.094	0.497	–	–	–
Student	0.765	0.613–0.953	0.017	0.849	0.673–1.072	0.169
Others	0.991	0.868–1.131	0.889	–	–	–
Region						
Northeast	Reference					
Southeast	1.049	0.896–1.229	0.550	–	–	–
Central	1.037	0.930–1.155	0.516	–	–	–
Northwest	0.945	0.887–1.007	0.082	0.933	0.876–0.994	**0.032**
Southwest	1.045	0.956–1.143	0.335	–	–	–
Partner's HIV status						
Not tested/Unknown	Reference					
Positive	1.091	1.000–1.190	0.051	1.027	0.925–1.141	0.613
Negative	1.031	0.947–1.123	0.481	–	–	–
STIs history						
Yes	0.975	0.903–1.054	0.525	–	–	–
No	1.045	0.973–1.123	0.225	–	–	–
Unknown	Reference					
Testing mode						
Opportunistic testing	Reference					
Voluntary testing	0.889	0.837–0.944	< 0.001	0.918	0.849–0.993	**0.033**
Compulsory testing	1.080	1.019–1.146	0.01	0.976	0.908–1.049	0.510
Similar symptoms in partner(s)						
No	Reference					
Yes	1.121	1.009–1.244	0.033	1.070	0.948–1.207	0.271
Sexual behavior						
Sexual contact with an HIV-positive partner	1.035	0.923–1.160	0.561	–	–	–
Non-marital commercial sex	1.010	0.939–1.087	0.787	–	–	–
Non-marital non-commercial heterosexual contact	1.041	0.972–1.116	0.253	–	–	–
MSM contact	0.914	0.862–0.970	0.003	1.015	0.947–1.089	0.671
Others	Reference					
No. of non-marital heterosexual partners	0.999	0.988–1.010	0.828	–	–	–
No. of homosexual partners	1.002	0.997–1.007	0.478	–	–	–

RR, relative risk; CI, confidence interval; STIs, sexually transmitted infections. Statistically significant results (*p* < 0.05) are shown in bold.

We further grouped patients into younger than 60 years and 60 years and older groups in Supplementary Table S5, which exhibited distinct profiles in the groups. Among 136 HIV patients aged 60 years or older, 119 (87.5%) were diagnosed late. The older group generally had lower levels of education compared with the younger group. Most older adults worked in manual and agricultural labor or were unemployed, while younger patients mostly worked in the services industry or sales. Notably, 62.5% older adults were tested compulsively while 49.3% younger patients were diagnosed voluntarily. Also, a higher proportion of older adults reported extramarital sex history, particularly commercial sex, compared with the younger group.

### 3.2 Subgroup analysis

Table 3 presents the stratified analysis results for each age group vs. testing behavior stratum. Relative risks (RRs) are reported with the overall study population as the reference stratum. The highest relative risk was observed in [Stratum 6] 1.097 (RR = 1.097, 95% PI: 1.013–1.174), whereas the lowest relative risk occurred in [Stratum 5] (RR = 0.89, 95% PI: 0.833–0.958). Both estimates remained stable before and after EB adjustment. Overall, the relative risk in the highest-risk stratum was approximately 23.3% greater than that in the lowest-risk stratum.

**Table 3:** Subgroup analysis by age group and testing behavior.

Stratum	Age group	Testing behavior	No. of late presenters	Total	Ra7w rate	Posterior rate	Posterior RR (95% PI)
1	≥60	Voluntary testing	16	17	0.941	0.813	1.065 (0.923–1.183)
2	50–59	Voluntary testing	40	50	0.800	0.783	1.026 (0.909–1.130)
3	40–49	Voluntary testing	59	80	0.738	0.747	0.979 (0.873–1.079)
4	30–39	Voluntary testing	135	182	0.742	0.746	0.977 (0.898–1.054)
5	< 30	Voluntary testing	209	313	0.668	0.680	0.890 (0.822–0.958)
6	≥60	Non-voluntary testing	103	119	0.866	0.838	1.097 (1.013–1.174)
7	50–59	Non-voluntary testing	96	115	0.835	0.815	1.068 (0.979–1.150)
8	40–49	Non-voluntary testing	127	152	0.836	0.819	1.074 (0.995–1.148)
9	30–39	Non-voluntary testing	146	179	0.816	0.805	1.055 (0.979–1.126)
10	< 30	Non-voluntary testing	141	197	0.716	0.725	0.949 (0.870–1.028)

RR, relative risk; PI, posterior interval.

### 3.3 Spatial autocorrelation analysis

Figure 1a shows the distribution of the proportion of late HIV diagnosis and the number of HIV cases in the 25 sub-districts. The sub-districts located in the northwest areas had a notably greater number of HIV cases but a lower late diagnosis proportion. The rest of the areas, especially the southeast areas, exhibit a low number of HIV cases but a relatively high proportion.

**Figure 1:**
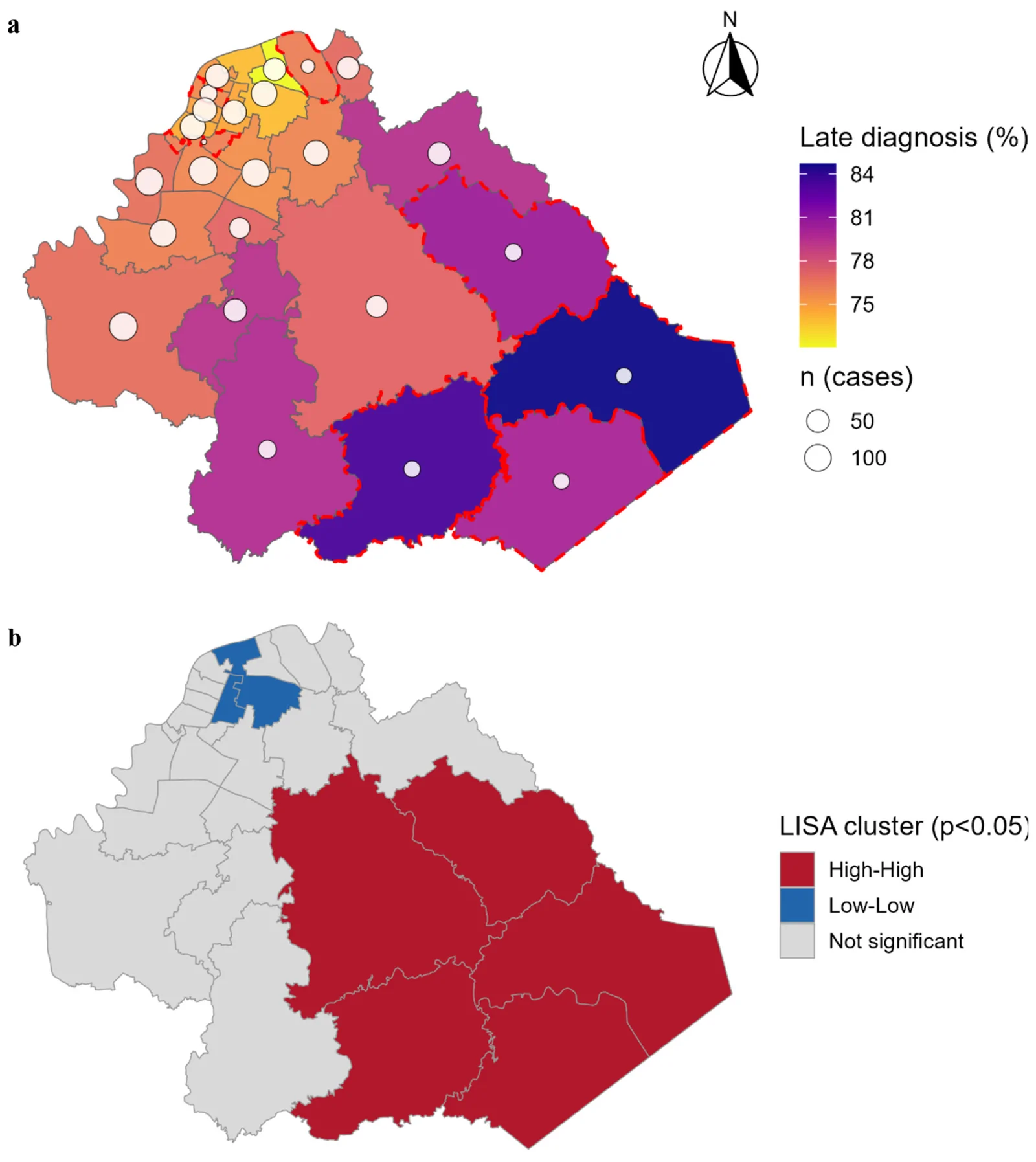
**(a)** Spatial distribution of late HIV diagnosis proportion and newly HIV cases across 25 sub-districts in Yinzhou (2002–2025); **(b)** Spatial clusters of late diagnosis proportion identified by LISA. **(a)** The color depth represents the proportion of late HIV diagnosis. The bubble size of each sub-district represents the total number of HIV cases. Sub-districts outlined by a red dashed line indicate fewer than 20 HIV cases were reported. **(b)** High-High clusters (red) indicate areas with consistently high proportion of late HIV diagnosis surrounded by similarly high proportion neighbors, whereas Low-Low clusters (blue) indicate areas with consistently low proportion. Not significant areas are shaded gray.

Result from the global Moran's I (0.65, *p* < 0.001) indicates strong spatial clustering of late diagnosis across sub-districts (Figure 1b). Local Moran's I further identified High-High clusters in the central and southeast areas and Low-Low clusters in the northwest areas, which is consistent with multivariate analysis. The rest of the areas were not significant in spatial analysis.

### 3.4 Survival analysis

There were 61 (4.3%) death cases during the study period, 58 were diagnosed late, and 3 were diagnosed not late, as shown in Supplementary Table S6. The median CD4 count at diagnosis was 206 cells/μl, and the median delay days was 8 for late presenters.

Kaplan–Meier survival curves showed that the survival probability of patients with late HIV diagnosis was significantly lower than non-late group (log-rank *p* = 0.002) throughout a 120-month follow-up (Supplementary Figure S2). Late HIV diagnosis was further found in Cox regression analysis associated with an increased risk of death (crude HR = 3.20, 95%CI: 1.14–9.03, *p*-value = 0.03), presented in Table 4. After adjustment for covariates, the association remained (HR = 3.03, 95% CI 1.04–8.84, *p* = 0.04), indicating that patients diagnosed late had a 3.04-fold higher hazard of death compared with those diagnosed not late.

**Table 4:** Cox regression analysis of late diagnosis to death.

Model	Variables	HR (95% CI)	*p*-value
Crude	**Late diagnosis**		
	Yes	3.20 (1.14–9.03)	0.03
	No	Reference	
Adjusted	**Late diagnosis**		
	Yes	3.03 (1.04–8.84)	0.04
	No	Reference	
	**Sex at birth**		
	Female	Reference	
	Male	1.08 (0.50–2.31)	0.85
	Educational level	0.77 (0.52–1.13)	0.18
	Voluntary testing	0.59 (0.30–1.17)	0.13
	Manual and agricultural workers	1.86 (1.06–3.29)	0.03

HR, hazard ratio; CI, confidence interval.

## 4 Discussion

The proportion of late diagnosis among PLWH is 76.4% in Yinzhou District, Ningbo, China, between 2002 and 2025. Using the same definition of late diagnosis of HIV, this proportion was higher than recent studies in China (8, 12, 17). Moreover, although the number of new HIV cases has generally declined over the past decade, the proportion of late diagnosis has overall increased, this disparity may lead to a misinterpretation of epidemic control and overlook the fact that large numbers of PLWH remain undiagnosed (22, 23). In addition, PLWH with late HIV diagnosis had a higher mortality risk than those with non-late HIV diagnosis. Therefore, there is an urgent need to develop new strategies to control and prevent late HIV diagnosis in the Yinzhou District.

Our findings suggest that the burden of late HIV diagnosis is shaped by overlapping socio-demographic and geographically patterned contextual factors. PLWH diagnosed at an older age were associated with a higher risk of late diagnosis in both Yinzhou and Ningbo, while PLWH tested voluntarily and living in the northwestern part of Yinzhou had a lower risk. Moreover, we identified a priority subgroup of PLWH aged 60 years or older who had non-voluntary testing behavior.

Spatial analysis further identified low-low clusters in the northwest of Yinzhou and high-high clusters in the southeast. The northwest region is entirely urbanized with adequate medical resources, while the southeast region is predominantly rural with almost no HIV-related medical resources. This observation is consistent with earlier studies, which reports that late HIV diagnosis is concentrated in rural areas and vice versa. A study in Tennessee, United States, found that the risk of late diagnosis increases with increasing level of rural; however, this association was no longer significant after adjusting system-level factors such as poverty, educational level and health insurance (24). On the other hand, a Swiss cohort study found a lower advanced presentation among people living in socioeconomic community (25). These findings suggest that late HIV diagnosis may be reduced by targeting for differences in socioeconomic status, healthcare accessibility, and risk awareness due to geographical variations. However, to explore potential mechanisms, determine the relationships between contextual factors and late diagnosis, and propose recommendations for resource allocation policy adjustments, future studies should measure or quantify indicators such as healthcare resources, testing coverage and socioeconomic development level.

Older age has become a widely recognized factor in late HIV diagnosis both domestically and internationally (8, 26). In this study, we extended this result by confirming the association of age and late HIV diagnosis in two independent cohorts. A recent epidemiology study showed the older population accounts for nearly half of new HIV cases in China, which may be due to low HIV testing uptake or engaging in high-risk sexual behavior, such as condomless commercial sex (27). Consistently, nearly half of the PLWH were tested voluntarily, and one-fifth of the PLWH had engaged in commercial sex. However, when grouped by age, the proportion of voluntary testing decreased; half of the older adults were tested compulsively, and one-third of the older adults reported having experienced commercial sex. The priority group we identified also supports age as a factor shaping health behavior. Studies have discovered a unique pattern of health-seeking behavior behind Chinese elders. For older Chinese adults living in rural areas, even when medical services are available, they are more likely to take empirical and informal self-treatment when symptoms arise. This is due to various reasons, including a perceived responsibility to the entire family to avoid consuming the family income or a fear that seeking medical resources would disrupt their normal daily routine (28, 29). Especially for older adults with a lower educational background, as they prioritize completing daily work, their self-perceived health state far outweighs objective tests unless other family members insist (30). Consequently, they may eventually be pressured by family members to undergo tests but miss the optimal opportunities to seek medical attention. These age-related barriers may also explain the spatial clustering observed in this study, especially if areas with a higher burden of late diagnosis include a larger rural older population. Therefore, applying interventions previously targeted to younger adults (e.g., education or expanding testing resources) may not be effective for older adults in this context.

Encouraging more individuals to take the initiative to test has always been a key focus of HIV prevention and control efforts. However, individual initiative is often constrained by social and structural factors. A study in Guangzhou, China revealed that the greater the distance to testing clinics, the lower the likelihood of being tested (31). Results from this study also indicated that imbalanced HIV-related medical resource distribution may hinder individual initiative to take the test. In this study, age, testing behavior, and spatial disparities jointly shape the epidemiology of late HIV diagnosis in Yinzhou. This overlap of factors is consistent with research on individual health behaviors, which are determined by multiple levels of factors, such as individual (e.g., physical and mental health status), interpersonal (e.g., relationship equity), institutional (e.g., sufficient resources), and structural (e.g., poverty) (32). Research based on this framework has made progress. A study based on multi-level data analysis has made a unique finding: females living in female-headed households had a higher risk of HIV than females living in male-dominant households (33). This finding provides valuable insights for developing targeted and personalized intervention plans at the local level. The feasibility of applying a multi-level factor framework to develop intervention programs was also confirmed. A five-year community-based experimental study exhibited that a multi-level factors-based intervention, including training female sex workers in negotiation skills with clients who are reluctant to use condoms, establishing community advisory committees to strengthen supervision, and providing 66 new VCT sites, can reduce HIV risk among female sex workers, as reflected in higher rates of HIV testing, condom use, and HIV prevention services utilization when compared with the standard care group (34). Therefore, systematic analysis is required to address underlying determinants of health behavior, helping prevent HIV infection and adverse outcomes among the older group in Yinzhou who faced disproportionate risk.

One limitation is that the definition of late HIV diagnosis in this study could classify patient in seroconversion as “late” due to their low CD4 count. Laboratory results should be collected, or clinical evidence should be considered in future studies to exclude patients in the acute phase. Second, routinely collected surveillance data did not include some important but not mandatory report items, such as the HIV status of sexual partners. These factors may be relevant to late HIV diagnosis. For example, a similar study found that having an HIV-positive sexual partner was associated with a higher risk of late diagnosis (35). The absence may therefore introduce residual confounding. Future studies should supplement surveillance data with questionnaires and medical records. Third, baseline characteristics differed between the derivation and validation cohorts, with SMDs >0.1 for several variables (e.g., age, education level). These imbalances limited direct comparability between cohorts, further validation in more diverse populations is needed. Finally, our study spans a 25-year period, during which public awareness and HIV testing strategies evolved substantially. While incorporating calendar year into our current analysis introduced model instability due to over-adjustment, future research should employ time-series modeling or time-stratified analyses to specifically investigate the epidemiological temporal trends impact the risk of late diagnosis.

In conclusion, this study highlights that age, testing behavior, and living areas are associated with late HIV diagnosis and suggests that individuals aged 60 years or older should be prioritized for promoting testing in the future. Late HIV diagnosis showed significant spatial clustering and was strongly associated with poorer survival outcomes in Yinzhou District. Together, these results underscore the importance of developing multi-level factors-based interventions that simultaneously address vulnerable populations and high-burden areas, with the ultimate goal of promoting earlier testing and timely treatment. In addition, policymakers should pay more attention to the high proportion of late HIV diagnosis, rather than solely focus on the number of new HIV cases to achieve the UNAIDS goal.

## Data Availability

The data analyzed in this study is subject to the following licenses/restrictions: The personal data from the Chinese Center for Disease Control and Prevention used in this study contain sensitive personal health information that cannot be shared publicly due to data protection and privacy regulations in China. Researchers can request access to de-identified data through the corresponding author, subject to approval from the local CDC and relevant ethics committees. Requests to access these datasets should be directed to Deguang Gong, 326225294@qq.com.
