# Supplementary material for: Spatial clustering and risk factors for late HIV diagnosis in the Yinzhou, China: a population-based retrospective cohort study
Source: Front Public Health. 2026 Oct 27;14:1888278. doi: 10.3389/fpubh.2026.1888278 (PMC13748371; doi:10.3389/fpubh.2026.1888278)
Supplement: Supplementary file 1 [file Data_Sheet_1.docx]

**Supplementary Table S1. Univariate analysis and multivariate analysis of factors associated with late HIV diagnosis (CD4 count < 200/µL) in 1404 HIV infected patients in Yinzhou district.**

| **Variables** | **Crude RR** | **95% CI** | ***p*-value** | **Adjusted RR** | **95% CI** | ***p*-value** |
| --- | --- | --- | --- | --- | --- | --- |
| **Age at diagnosis** | 1.017 | 1.012-1.021 | <0.001 | 1.013 | 1.007- 1.020 | **<0.001** |
| **Driving distance to clinic (km)** | 0.998 | 0.987-1.009 | 0.773 |  |  |  |
| **Sex at birth** |  |  |  |  |  |  |
| Female | Reference |  |  |  |  |  |
| Male | 1.016 | 0.832-1.240 | 0.879 |  |  |  |
| **Marriage status** |  |  |  |  |  |  |
| Unknown | Reference |  |  |  |  |  |
| Married or partnered | 1.277 | 1.072-1.043 | 0.003 | 0.869 | 0.700-1.078 | 0.201 |
| Divorced or widowed | 1.299 | 1.103-1.529 | 0.002 | 0.992 | 0.789-1.247 | 0.946 |
| **Educational level** | 0.881 | 0.831-0.935 | <0.001 | 1.042 | 0.967-1.123 | 0.284 |
| **Occupation** |  |  |  |  |  |  |
| Services and sales workers | 0.792 | 0.685-0.915 | 0.002 | 0.860 | 0.697-1.062 | 0.161 |
| Government employees and professionals | Reference |  |  |  |  |  |
| Manual or Agricultural workers | 1.236 | 1.062-1.438 | 0.006 | 0.977 | 0.777-1.229 | 0.845 |
| Fishery workers | 1.334 | 0.598-2.978 | 0.482 |  |  |  |
| Not in the labor force | 1.189 | 1.025-1.379 | 0.022 | 0.905 | 0.725-1.128 | 0.373 |
| Student | 0.418 | 0.229-0.764 | 0.005 | 0.523 | 0.277-0.988 | **0.046** |
| Others | 1.055 | 0.792-1.405 | 0.716 |  |  |  |
| **Region** |  |  |  |  |  |  |
| Northeast | 0.937 | 0.675-1.303 | 0.7 |  |  |  |
| Southeast | Reference |  |  |  |  |  |
| Central | 0.98 | 0.746-1.287 | 0.886 |  |  |  |
| Northwest | 1.033 | 0.881-1.211 | 0.691 | 0.933 | 0.876-0.994 | **0.032** |
| Southwest | 1.023 | 0.820-1.276 | 0.843 |  |  |  |
| **Partner’s HIV status** |  |  |  |  |  |  |
| Not tested/Unknown | Reference |  |  |  |  |  |
| Positive | 0.910 | 0.759-1.091 | 0.307 |  |  |  |
| Negative | 1.127 | 0.954-1.331 | 0.159 | 1.162 | 0.954-1.415 | 0.135 |
| **STIs history** |  |  |  |  |  |  |
| Yes | 0.975 | 0.903-1.054 | 0.525 |  |  |  |
| No | 1.299 | 1.103-1.529 | 0.002 | 0.992 | 0.789-1.247 | 0.946 |
| Unknown | Reference |  |  |  |  |  |
| **Testing Mode** |  |  |  |  |  |  |
| Opportunistic testing | Reference |  |  |  |  |  |
| Voluntary testing | 0.889 | 0.837-0.944 | <0.001 | 0.918 | 0.849-0.993 | **0.033** |
| Compulsory testing | 1.080 | 1.019-1.146 | 0.01 | 0.976 | 0.908-1.049 | 0.510 |
| **Similar symptoms in partner(s)** | |  |  |  |  |  |
| No | Reference |  |  |  |  |  |
| Yes | 1.121 | 1.009-1.244 | 0.033 | 1.070 | 0.948-1.207 | 0.271 |
| **Sexual behavior** |  |  |  |  |  |  |
| Sexual contact with an HIV-positive partner | 1.035 | 0.923-1.160 | 0.561 |  |  |  |
| Non-marital commercial sex | 1.010 | 0.939-1.087 | 0.787 |  |  |  |
| Non-marital non-commercial heterosexual contact | 1.041 | 0.972-1.116 | 0.253 |  |  |  |
| MSM contact | 0.914 | 0.862-0.970 | 0.003 | 1.015 | 0.947-1.089 | 0.671 |
| Others | Reference |  |  |  |  |  |
| **No. of non-marital heterosexual partners** | 0.999 | 0.988-1.010 | 0.828 |  |  |  |
| **No. of homosexual partners** | 1.002 | 0.997-1.007 | 0.478 |  |  |  |

Abbreviations: RR – relative risk; CI – confidence interval; STIs – Sexually transmitted infections.

**Supplementary Table S2. Confounder analysis.**

| **Variables** | **Association with exposure** | ***p*-value** | **Association with outcome** | ***p*-value** | **delta** | **Crude HR of exposure** | **Adjusted HR of exposure** | **Candidate by p-value** | **Candidate by delta** |
| --- | --- | --- | --- | --- | --- | --- | --- | --- | --- |
| **Age at diagnosis** | logit (numeric) | 0.0 | coxPH (Wald) | 0.0 | -33.87 | 5.2 | 3.44 | TRUE | TRUE |
| **Single** | logit (categorical, LR test) | 0.0 | coxPH (Wald) | 0.0 | -18.18 | 5.2 | 4.25 | TRUE | TRUE |
| **Educational level** | logit (categorical, LR test) | 0.0 | coxPH (LR, categorical) | 0.0 | -22.46 | 5.2 | 4.03 | TRUE | TRUE |
| **Voluntary testing** | logit (categorical, LR test) | 0.0 | coxPH (Wald) | 0.0 | -11.61 | 5.2 | 4.59 | TRUE | TRUE |
| **Manual and agricultural workers** | logit (categorical, LR test) | 0.0 | coxPH (Wald) | 0.0 | -12.53 | 5.2 | 4.55 | TRUE | TRUE |
| **Divorced or widowed** | logit (categorical, LR test) | 0.0 | coxPH (Wald) | 0.03 | -4.57 | 5.2 | 4.96 | TRUE | FALSE |
| **Heterosexual contact** | logit (categorical, LR test) | 0.0 | coxPH (Wald) | 0.0 | -8.76 | 5.2 | 4.74 | TRUE | FALSE |
| **Same-sex contact** | logit (categorical, LR test) | 0.0 | coxPH (Wald) | 0.0 | -9.78 | 5.2 | 4.69 | TRUE | FALSE |
| **MSM contact** | logit (categorical, LR test) | 0.0 | coxPH (Wald) | 0.0 | -9.66 | 5.2 | 4.69 | TRUE | FALSE |
| **Compulsory testing** | logit (categorical, LR test) | 0.01 | coxPH (Wald) | 0.04 | -3.56 | 5.2 | 5.01 | TRUE | FALSE |
| **Married or partnered** | logit (categorical, LR test) | 0.01 | coxPH (Wald) | 0.0 | -7.32 | 5.2 | 4.82 | TRUE | FALSE |
| **Opportunistic testing** | logit (categorical, LR test) | 0.05 | coxPH (Wald) | 0.0 | -3.24 | 5.2 | 5.03 | TRUE | FALSE |
| **Services and sales workers** | logit (categorical, LR test) | 0.07 | coxPH (Wald) | 0.01 | -3.82 | 5.2 | 5.0 | TRUE | FALSE |
| **No similar symptoms occurred in partner(s)** | logit (categorical, LR test) | 0.07 | coxPH (Wald) | 0.0 | -7.06 | 5.2 | 4.83 | TRUE | FALSE |
| **similar symptoms occurred in partner(s)** | logit (categorical, LR test) | 0.07 | coxPH (Wald) | 0.0 | -7.06 | 5.2 | 4.83 | TRUE | FALSE |
| **Student** | logit (categorical, LR test) | 0.0 | coxPH (Wald) | 0.78 | -0.06 | 5.2 | 5.19 | FALSE | FALSE |
| **Drug injection** | logit (categorical, LR test) | 0.03 | coxPH (Wald) | 1.0 | -1.03 | 5.2 | 5.14 | FALSE | FALSE |
| **Positive sexual partner** | logit (categorical, LR test) | 0.08 | coxPH (Wald) | 0.19 | -1.65 | 5.2 | 5.11 | FALSE | FALSE |
| **Unknown HIV status in partner** | logit (categorical, LR test) | 0.08 | coxPH (Wald) | 0.78 | 0.56 | 5.2 | 5.23 | FALSE | FALSE |
| **No. of same sex partners** | logit (numeric) | 0.09 | coxPH (Wald) | 0.97 | 0.14 | 3.65 | 3.65 | FALSE | FALSE |
| **Northwest** | logit (categorical, LR test) | 0.09 | coxPH (Wald) | 0.63 | 0.86 | 5.2 | 5.24 | FALSE | FALSE |
| **Unknown STIs history** | logit (categorical, LR test) | 0.15 | coxPH (Wald) | 0.74 | 0.2 | 5.2 | 5.21 | FALSE | FALSE |
| **Vertical transmission** | logit (categorical, LR test) | 0.2 | coxPH (Wald) | 0.04 | -1.13 | 5.2 | 5.14 | FALSE | FALSE |
| **No STIs history** | logit (categorical, LR test) | 0.21 | coxPH (Wald) | 0.16 | -1.16 | 5.2 | 5.14 | FALSE | FALSE |
| **Non-marital sexual contact** | logit (categorical, LR test) | 0.27 | coxPH (Wald) | 0.11 | -0.22 | 5.2 | 5.19 | FALSE | FALSE |
| **Blood transfusion** | logit (categorical, LR test) | 0.3 | coxPH (failed) | NA | NA | NA | NA | FALSE | FALSE |
| **Northeast** | logit (categorical, LR test) | 0.36 | coxPH (Wald) | 0.88 | 0.01 | 5.2 | 5.2 | FALSE | FALSE |
| **Southwest** | logit (categorical, LR test) | 0.36 | coxPH (Wald) | 0.41 | -0.43 | 5.2 | 5.17 | FALSE | FALSE |
| **Unknown marital status** | logit (categorical, LR test) | 0.42 | coxPH (Wald) | 1.0 | -0.01 | 5.2 | 5.2 | FALSE | FALSE |
| **Other transmission route** | logit (categorical, LR test) | 0.46 | coxPH (Wald) | 1.0 | 0.16 | 5.2 | 5.21 | FALSE | FALSE |
| **Blood collection** | logit (categorical, LR test) | 0.46 | coxPH (Wald) | 1.0 | 0.16 | 5.2 | 5.21 | FALSE | FALSE |
| **Female** | logit (categorical, LR test) | 0.49 | coxPH (Wald) | 0.15 | -0.72 | 5.2 | 5.16 | FALSE | FALSE |
| **Male** | logit (categorical, LR test) | 0.49 | coxPH (Wald) | 0.15 | -0.72 | 5.2 | 5.16 | FALSE | FALSE |
| **Negative sexual partner** | logit (categorical, LR test) | 0.49 | coxPH (Wald) | 0.14 | 0.68 | 5.2 | 5.23 | FALSE | FALSE |
| **Had STIs history** | logit (categorical, LR test) | 0.52 | coxPH (Wald) | 0.06 | -1.45 | 5.2 | 5.12 | FALSE | FALSE |
| **Central** | logit (categorical, LR test) | 0.53 | coxPH (Wald) | 0.28 | 0.58 | 5.2 | 5.23 | FALSE | FALSE |
| **Not in the labor force** | logit (categorical, LR test) | 0.55 | coxPH (Wald) | 0.32 | 0.42 | 5.2 | 5.22 | FALSE | FALSE |
| **Southeast** | logit (categorical, LR test) | 0.57 | coxPH (Wald) | 0.99 | -0.12 | 5.2 | 5.19 | FALSE | FALSE |
| **Sexual contact with an HIV-positive partner** | logit (categorical, LR test) | 0.58 | coxPH (Wald) | 0.0 | -2.65 | 5.2 | 5.06 | FALSE | FALSE |
| **Fishery workers** | logit (categorical, LR test) | 0.59 | coxPH (Wald) | 0.18 | 1.11 | 5.2 | 5.25 | FALSE | FALSE |
| **Government Employees and Professionals** | logit (categorical, LR test) | 0.64 | coxPH (Wald) | 0.15 | 0.31 | 5.2 | 5.21 | FALSE | FALSE |
| **No. of non-marital heterosexual partners** | logit (numeric) | 0.65 | coxPH (Wald) | 0.08 | 3.64 | 3.65 | 3.79 | FALSE | FALSE |
| **Free testing** | logit (categorical, LR test) | 0.7 | coxPH (Wald) | 0.1 | -0.77 | 5.2 | 5.16 | FALSE | FALSE |
| **Non-marital commercial sexual contact** | logit (categorical, LR test) | 0.79 | coxPH (Wald) | 0.17 | -0.3 | 5.2 | 5.18 | FALSE | FALSE |
| **Driving distance** | logit (numeric) | 0.83 | coxPH (Wald) | 0.88 | -0.05 | 5.2 | 5.19 | FALSE | FALSE |
| **Other occupations** | logit (categorical, LR test) | 0.99 | coxPH (Wald) | 0.69 | 0.21 | 5.2 | 5.21 | FALSE | FALSE |

Abbreviations: LR – likelihood ratio; HR – Hazard ratio; PH – proportional hazard; STIs – sexually transmitted infections; MSM – men who had sex with men.

Potential confounders were identified through a two-step procedure. First, each variable was tested for association with both the exposure and outcome using appropriate statistical methods. Associations with the exposure were examined using logistic regression, applying the Wald test for numeric variables and the likelihood ratio (LR) test for categorical variables. Associations with the outcome were assessed using univariate Cox proportional hazards models, using the Wald test for numeric and binary variables and the LR test for categorical variables.

Second, a change-in-estimate criterion was applied by comparing the crude and adjusted hazard ratios (HRs). The percentage change in HR (ΔHR%) was calculated as:

$$\Delta HR\%=\frac{{HR}_{adjusted} - {HR}_{crude}}{{HR}_{crude}}\times100$$

Variables producing a change of ≥10% were considered potential confounders and included in the adjusted model.

Based on this criterion, five variables were identified as potential confounders, of which four were included in the adjusted model. Age was not adjusted for, as it was set as the time scale of the model.

**Supplementary Table S3. Proportional hazards test.**

| **Variables** | **Initial PH test** | | | **Stratified PH test** | | |
| --- | --- | --- | --- | --- | --- | --- |
|  | **Chi-square** | **df** | ***p*-value** | **Chi-square** | **df** | ***p*-value** |
| **LD** | 0.33 | 1 | 0.57 | 0.33 | 1 | 0.57 |
| **Sex at birth** | 0.15 | 1 | 0.69 | 0.10 | 1 | 0.76 |
| **Single** | 6.74 | 1 | 0.01 | 1.80 | 1 | 0.18 |
| **Educational level** | 1.67 | 1 | 0.20 | Stratified |  |  |
| **Voluntary testing** | 0.93 | 1 | 0.34 | 0.99 | 1 | 0.32 |
| **Manual and agricultural workers** | 0.01 | 1 | 0.94 | <0.01 | 1 | 0.98 |
| **Global** | 11.08 | 6 | 0.05 | 2.97 | 5 | 0.56 |

Abbreviations: PH – proportional hazard; LD – late diagnosis; df – degree of freedom.

PH were assessed with Schoenfeld residual tests. In initial PH diagnostics, marital status (single) showed evidence of non-proportional hazards (Schoenfeld test p = 0.01; GLOBAL p = 0.05). The model was therefore refitted stratifying by marital status. After stratification, no violations of the PH assumption were observed (all covariate-specific p ≥ 0.05; GLOBAL p = 0.61). As stratification absorbs differences in baseline hazards, no hazard ratio is presented for marital status.

**Supplementary Table S4. Characteristics of 324 HIV infected patients in Ningbo (2007-2025), grouped by late diagnosis and non-late diagnosis of HIV.**

| **Characteristics** | **Overall**  N = 324 | **LD**  N = 249 (76.9%) | **Non-LD**  N = 75  (23.1%) |
| --- | --- | --- | --- |
| **Age at diagnosis**, Median [Q1, Q3] | 47.0 [36.0, 60.0] | 49.0 [38.0, 63.0] | 44.0 [33.5, 52.0] |
| **No. of non-marital heterosexual partners**, Median  [Q1, Q3] | 3.0 [1.0, 5.0] | 3.0 [1.0, 5.0] | 3.0 [2.0, 4.5] |
| **No. of same sex partners**, Median  [Q1, Q3] | 4.0 [2.0, 6.0] | 4.0 [2.0, 6.0] | 4.0 [2.0, 6.2] |
| **Sex at birth**, n (%) |  |  |  |
| Female | 68 (21.0%) | 57 (22.9%) | 11 (14.7%) |
| Male | 256 (79.0%) | 192 (77.1%) | 64 (85.3%) |
| **Marriage status**, n (%) | |  |  |
| Single | 83 (25.6%) | 56 (22.5%) | 27 (36.0%) |
| Married/partnered | 161 (49.7%) | 135 (54.2%) | 26 (34.7%) |
| Divorced/widowed | 78 (24.1%) | 56 (22.5%) | 22 (29.3%) |
| Unknown | 2 (0.6%) | 2 (0.8%) | 0 (0.0%) |
| **Education**, n (%) |  |  |  |
| Primary or below | 103 (31.8%) | 87 (34.9%) | 16 (21.3%) |
| Lower secondary education | 102 (31.5%) | 75 (30.1%) | 27 (36.0%) |
| Upper secondary education | 70 (21.6%) | 50 (20.1%) | 20 (26.7%) |
| Tertiary education | 49 (15.1%) | 37 (14.9%) | 12 (16.0%) |
| **Occupation**, n (%) |  |  |  |
| Services and sales workers | 41 (12.7%) | 29 (11.6%) | 12 (16.0%) |
| Government employees and professionals | 9 (2.8%) | 8 (3.2%) | 1 (1.3%) |
| Manual and agricultural workers | 158 (48.8%) | 128 (51.4%) | 30 (40.0%) |
| Fishery workers | 4 (1.2%) | 2 (0.8%) | 2 (2.7%) |
| Not in labor force | 9 (2.8%) | 8 (3.2%) | 1 (1.3%) |
| Student | 4 (1.2%) | 2 (0.8%) | 2 (2.7%) |
| Others | 9 (2.8%) | 6 (2.4%) | 3 (4.0%) |
| **Partner’s HIV status**, n (%) | |  |  |
| Negative | 69 (21.3%) | 53 (21.3%) | 16 (21.3%) |
| Positive | 41 (12.7%) | 38 (15.3%) | 3 (4.0%) |
| Not tested/Unknown | 214 (66.0%) | 158 (63.5%) | 56 (74.7%) |
| **STIs history**, n (%) |  |  |  |
| Yes | 59 (18.2%) | 43 (17.3%) | 16 (21.3%) |
| No | 227 (70.1%) | 179 (71.9%) | 48 (64.0%) |
| Unknown | 38 (11.7%) | 27 (10.8%) | 11 (14.7%) |
| **Testing behavior**, n (%) |  |  |  |
| Voluntary testing | 92 (28.4%) | 65 (26.1%) | 27 (36.0%) |
| Compulsory testing | 162 (50.0%) | 127 (51.0%) | 35 (46.7%) |
| Opportunistic testing | 70 (21.6%) | 57 (22.9%) | 13 (17.3%) |
| **Sexual behavior**^1^**, n (%)** |  |  |  |
| Sexual contact with an HIV-positive partner | 26 (8%) | 23 (9.2%) | 3 (4%) |
| Non-marital commercial sexual contact | 79 (24.4%) | 65 (26.1%) | 14 (18.7%) |
| Non-marital sexual contact | 72 (22.2%) | 55 (22.1%) | 17 (22.7%) |
| MSM contact | 124 (38.3%) | 84 (33.7%) | 40 (53.3%) |
| Others | 16 (4.9%) | 13 (5.2%) | 3 (4%) |

^1^Sexual behavior was a multiple-choices question, and those who responded “Yes” were included in the table.

Abbreviations: LD – late diagnosis; STIs – Sexually transmitted infections.

**Supplementary Table S5. Univariate analysis and multivariate analysis of factors associated with late HIV diagnosis in 324 HIV infected patients in Ningbo (2007-2025).**

| Variable | Crude RR | 95% CI | *p*-value | Adjusted RR | 95% CI | *p*-value |
| --- | --- | --- | --- | --- | --- | --- |
| **Age at diagnosis** | 1.007 | 1.004-1.011 | < 0.001 | 1.007 | 1.002-1.012 | 0.004 |
| **Sex at birth** |  |  |  |  |  |  |
| Female | Reference |  |  |  |  |  |
| Male | 0.895 | 0.789-1.015 | 0.084 | 0.942 | 0.807-1.100 | 0.450 |
| **Marriage status** |  |  |  |  |  |  |
| Unknown | Reference |  |  |  |  |  |
| Married or partnered | 1.199 | 1.062-1.354 | 0.003 | 1.146 | 0.980-1.340 | 0.088 |
| **Educational level** | 0.953 | 0.901-1.009 | 0.099 | 1.054 | 0.975-1.140 | 0.187 |
| **Occupation** |  |  |  |  |  |  |
| Services and sales workers | 0.910 | 0.740-1.119 | 0.370 |  |  |  |
| Government employees and professionals | Reference |  |  |  |  |  |
| Manual and agricultural workers | 1.111 | 0.986-1.253 | 0.083 | 1.106 | 0.966-1.267 | 0.145 |
| Fishery workers | 0.648 | 0.243-1.729 | 0.386 |  |  |  |
| Not in labor force | 1.162 | 0.915-1.475 | 0.219 |  |  |  |
| Student | 0.648 | 0.243-1.729 | 0.386 |  |  |  |
| Others | 0.864 | 0.542-1.377 | 0.539 |  |  |  |
| **Partner’s HIV status** |  |  |  |  |  |  |
| Not tested/Unknown | Reference |  |  |  |  |  |
| Positive | 1.243 | 1.114-1.387 | 0.001 | 1.044 | 0.931-1.170 | 0.465 |
| Negative | 0.999 | 0.864-1.157 | 0.993 |  |  |  |
| **STIs history** |  |  |  |  |  |  |
| Yes | 0.938 | 0.792-1.110 | 0.453 |  |  |  |
| No | 1.093 | 0.949-1.258 | 0.217 |  |  |  |
| Unknown | Reference |  |  |  |  |  |
| **Testing behavior** |  |  |  |  |  |  |
| Opportunistic testing | 1.077 | 0.944-1.229 | 0.269 |  |  |  |
| Voluntary testing | 0.891 | 0.769-1.032 | 0.124 |  |  |  |
| Compulsory testing | Reference |  |  |  |  |  |
| **Sexual behavior** |  |  |  |  |  |  |
| Sexual contact with an HIV-positive partner | 1.166 | 1.001-1.359 | 0.048 | 1.049 | 0.896-1.228 | 0.556 |
| Non-marital commercial sex | 1.096 | 0.967-1.242 | 0.153 |  |  |  |
| Non-marital non-commercial heterosexual contact | 0.992 | 0.858-1.147 | 0.916 |  |  |  |
| MSM contact | 0.821 | 0.716-0.942 | 0.005 | 0.893 | 0.749-1.065 | 0.207 |
| Others | Reference |  |  |  |  |  |
| **No. of non-marital heterosexual partners** | 0.990 | 0.972-1.009 | 0.290 |  |  |  |
| **No. of same sex partners** | 0.988 | 0.972-1.004 | 0.152 |  |  |  |

**Supplementary Table S6. Characteristics of 1404 HIV infected patients in the Yinzhou district (2002-2025), grouped by age and late diagnosis.**

| **Characteristics** | **Age < 60** | | | **Age ≥ 60** | | |
| --- | --- | --- | --- | --- | --- | --- |
|  | **Total**  N = 1268 | **LD**  N = 953 (75.2%) | **Non-LD**  N = 315  (24.8%) | **Total**  N = 136 | **LD**  N = 119 (87.5%) | **Non-LD**  N = 17 (12.5%) |
| **Driving distance to clinic (km)**, Median  [Q1, Q3] | 6.0 [3.4, 9.2] | 5.9[3.3, 9.3] | 6.0[3.4, 9.0] | 8.2 [5.2, 14.1] | 7.8[4.8, 13.5] | 9.3[8.2, 16.6] |
| **No. of non-marital heterosexual partners,** Median  [Q1, Q3] | 2.0 [2.0, 4.0] | 2.0[1.0, 4.0] | 2.0[2.0, 4.2] | 2.0 [2.0, 3.0] | 2.0[2.0, 3.0] | 2.0[2.0, 3.8] |
| **No. of same-sex partners,** Median  [Q1, Q3] | 5.0 [3.0, 10.0] | 5.0[3.0, 10.0] | 5.0[3.0, 8.0] | 5.0 [3.2, 10.0] | 5.0[3.2, 10.0] | 0 |
| **Sex at birth**, n (%) |  |  |  |  |  |  |
| Female | 165 (13.0%) | 130 (13.6%) | 35 (11.1%) | 24 (17.6%) | 18 (15.1%) | 6 (35.3%) |
| Male | 1103 (87.0%) | 823 (86.4%) | 280 (88.9%) | 112 (82.4%) | 101 (84.9%) | 11 (64.7%) |
| **Marriage status**, n (%) |  |  |  |  |  |  |
| Single | 664 (52.4%) | 471 (49.4%) | 193 (61.3%) | 136 (100.0%) | 0 (0.0%) | 0 (0.0%) |
| Married/partnered | 426 (33.6%) | 335 (35.2%) | 91 (28.9%) | 102 (75.0%) | 87 (73.1%) | 15 (88.2%) |
| Divorced/widowed | 176 (13.9%) | 146 (15.3%) | 30 (9.5%) | 34 (25.0%) | 32 (26.9%) | 2 (11.8%) |
| Unknown | 2 (0.2%) | 1 (0.1%) | 1 (0.3%) | 136 (100.0%) | 0 (0.0%) | 0 (0.0%) |
| **Education**, n (%) |  |  |  |  |  |  |
| Primary or below | 136 (10.7%) | 115 (12.1%) | 21 (6.7%) | 87 (64.0%) | 75 (63.0%) | 12 (70.6%) |
| Lower secondary education | 359 (28.3%) | 281 (29.5%) | 78 (24.8%) | 26 (19.1%) | 21 (17.6%) | 5 (29.4%) |
| Upper secondary education | 256 (20.2%) | 192 (20.1%) | 64 (20.3%) | 14 (10.3%) | 14 (11.8%) | 0 (0.0%) |
| Tertiary education | 517 (40.8%) | 365 (38.3%) | 152 (48.3%) | 9 (6.6%) | 9 (7.6%) | 0 (0.0%) |
| **Occupation**, n (%) |  |  |  |  |  |  |
| Services and Sales workers | 543 (42.8%) | 401 (42.1%) | 142 (45.1%) | 10 (7.4%) | 7 (5.9%) | 3 (17.6%) |
| Government employees and Professionals | 88 (6.9%) | 69 (7.2%) | 19 (6.0%) | 0 (0.0%) | 0 (0.0%) | 0 (0.0%) |
| Manual and Agricultural workers | 239 (18.8%) | 193 (20.3%) | 46 (14.6%) | 48 (35.3%) | 44 (37.0%) | 4 (23.5%) |
| Fishery workers | 6 (0.5%) | 4 (0.4%) | 2 (0.6%) | 0 (0.0%) | 0 (0.0%) | 0 (0.0%) |
| Not in labor force | 254 (20.0%) | 190 (19.9%) | 64 (20.3%) | 76 (55.9%) | 66 (55.5%) | 10 (58.8%) |
| Student | 56 (4.4%) | 33 (3.5%) | 23 (7.3%) | 0 (0.0%) | 0 (0.0%) | 0 (0.0%) |
| Others | 75 (5.9%) | 57 (6.0%) | 18 (5.7%) | 1 (0.7%) | 1 (0.8%) | 0 (0.0%) |
| **Orientation**, n (%) |  |  |  |  |  |  |
| Northeast | 63 (5.0%) | 51 (5.4%) | 12 (3.8%) | 5 (3.7%) | 4 (3.4%) | 1 (5.9%) |
| Southeast | 35 (2.8%) | 27 (2.8%) | 8 (2.5%) | 5 (3.7%) | 5 (4.2%) | 0 (0.0%) |
| Central | 89 (7.0%) | 69 (7.2%) | 20 (6.3%) | 6 (4.4%) | 6 (5.0%) | 0 (0.0%) |
| Northwest | 947 (74.7%) | 700 (73.5%) | 247 (78.4%) | 113 (83.1%) | 98 (82.4%) | 15 (88.2%) |
| Southwest | 134 (10.6%) | 106 (11.1%) | 28 (8.9%) | 7 (5.1%) | 6 (5.0%) | 1 (5.9%) |
| **Partner’s HIV status**, n (%) | |  |  |  |  |  |
| Positive | 96 (7.6%) | 79 (8.3%) | 17 (5.4%) | 25 (18.4%) | 21 (17.6%) | 4 (23.5%) |
| Negative | 145 (11.4%) | 111 (11.6%) | 34 (10.8%) | 22 (16.2%) | 20 (16.8%) | 2 (11.8%) |
| Not tested/Unknown | 1027 (81.0%) | 763 (80.1%) | 264 (83.8%) | 89 (65.4%) | 78 (65.5%) | 11 (64.7%) |
| **STIs history**, n (%) |  |  |  |  |  |  |
| Yes | 245 (19.3%) | 180 (18.9%) | 65 (20.6%) | 17 (12.5%) | 16 (13.4%) | 1 (5.9%) |
| No | 957 (75.5%) | 727 (76.3%) | 230 (73.0%) | 111 (81.6%) | 97 (81.5%) | 14 (82.4%) |
| Unknown | 63 (5.0%) | 43 (4.5%) | 20 (6.3%) | 8 (5.9%) | 6 (5.0%) | 2 (11.8%) |
| **Testing behavior**, n (%) |  |  |  |  |  |  |
| Compulsory testing | 383 (30.2%) | 301 (31.6%) | 82 (26.0%) | 85 (62.5%) | 75 (63.0%) | 10 (58.8%) |
| Opportunistic testing | 260 (20.5%) | 209 (21.9%) | 51 (16.2%) | 34 (25.0%) | 28 (23.5%) | 6 (35.3%) |
| Voluntary testing | 625 (49.3%) | 443 (46.5%) | 182 (57.8%) | 17 (12.5%) | 16 (13.4%) | 1 (5.9%) |
| **Similar symptoms in partner(s), n (%)** | | |  |  |  |  |
| No | 1211 (95.5%) | 905 (95.0%) | 306 (97.1%) | 126 (92.6%) | 110 (92.4%) | 16 (94.1%) |
| Yes | 57 (4.5%) | 48 (5.0%) | 9 (2.9%) | 10 (7.4%) | 9 (7.6%) | 1 (5.9%) |
| **Sexual behavior**^1^**, n (%)** |  |  |  |  |  |  |
| Sexual contact with an HIV-positive partner | 64 (5.0%) | 50 (5.2%) | 14 (4.4%) | 21 (15.4%) | 17 (14.3%) | 4 (23.5%) |
| Non-marital commercial sexual contact | 200 (15.8%) | 149 (15.6%) | 51 (16.2%) | 65 (47.8%) | 55 (46.2%) | 10 (58.8%) |
| Non-marital sexual contact | 249 (19.6%) | 194 (20.4%) | 55 (17.5%) | 30 (22.1%) | 26 (21.8%) | 4 (23.5%) |
| MSM contact | 659 (52.0%) | 476 (49.9%) | 183 (58.1%) | 14 (10.3%) | 14 (11.8%) | 0 (0.0%) |
| Others | 16 (1.3%) | 13 (1.4%) | 3 (1.0%) | 1 (0.7%) | 1 (0.8%) | 0 (0.0%) |
| **Possible transmission route, n (%)** | | |  |  |  |  |
| Heterosexual transmission | 600 (47.3%) | 471 (49.4%) | 129 (41.0%) | 122 (89.7%) | 105 (88.2%) | 17 (100.0%) |
| Same-sex transmission^2^ | 657 (51.8%) | 474 (49.7%) | 183 (58.1%) | 14 (10.3%) | 14 (11.8%) | 0 (0.0%) |
| Others | 11 (0.9%) | 8 (0.8%) | 3 (1.0%) | 0 (0.0%) | 0 (0.0%) | 0 (0.0%) |

^1^Sexual behavior was a multiple-choices question, and those who responded “Yes” were included in the table.

^2^Only male responded to this information.

Abbreviations: LD – late diagnosis; STIs – sexually transmitted infections.

**Supplementary Table S7. Follow-up outcomes.**

| **Characteristics** | **Overall**  **N=1404** | **LD**  **N=1072** | **Non-LD**  **N=332** | ***p*-value** |
| --- | --- | --- | --- | --- |
| **Survival status**, n (%) |  |  |  | <0.001 |
| Alive | 1,343 (96%) | 1,014 (95%) | 329 (99%) |  |
| Dead | 61 (4.3%) | 58 (5.4%) | 3 (0.9%) |  |
| **AIDS-defining events**, n (%) |  |  |  | <0.001 |
| Yes | 81 (5.8%) | 81 (7.6%) | 0 (0%) |  |
| No | 1,323 (94%) | 991 (92%) | 332 (100%) |  |
| **Initial CD4 count (cells/μL)**, Median (Q1, Q3) | 242.5  (149.5, 343.0) | 206.0  (114.5, 268.5) | 435.0  (388.5, 506.0) | <0.001 |
| **Initial viral load (copies/mL)**, Median (Q1, Q3)^1^ | 20.0  (0.0, 23200.0) | 20.0  (0.0, 31,200.0) | 0.0  (0.0, 10,400.0) | 0.062 |
| **Delay diagnosis days**, Median (Q1, Q3) | 7.0  (3.0, 16.0) | 8.0  (3.0, 20.5) | 6.0  (3.0, 9.0) | <0.001 |
| **Loss to follow-up**^2^, n (%) |  |  |  | 0.029 |
| Yes | 333 (24%) | 239 (22%) | 94 (28%) |  |
| No | 1,071 (76%) | 833 (78%) | 238 (72%) |  |
| **Loss to follow-up rate (%)**^3^ |  |  |  | 0.073 |
| 1-20 | 316 (23%) | 227 (21%) | 89 (27%) |  |
| >20 | 17 (1.2%) | 12 (1.1%) | 5 (1.5%) |  |
| No | 1,066 (76%) | 830 (78%) | 236 (72%) |  |
| **WHO clinical stage**, n (%) |  |  |  | <0.001 |
| I | 698 (50%) | 523 (49%) | 175 (53%) |  |
| II | 512 (36%) | 368 (34%) | 144 (43%) |  |
| III | 137 (9.8%) | 124 (12%) | 13 (3.9%) |  |
| IV | 57 (4.1%) | 57 (5.3%) | 0 (0%) |  |

^1^ 22 patients did not test viral load when diagnosed.

^2^ As of the time of data query, five patients had not started the second follow-up.

^3^ The rate of loss to follow-up was calculated by dividing the number of losses to follow-up by the total number of follow-up visits.

Abbreviations: LD – late diagnosis; AIDS – acquired immunodeficiency syndrome; WHO – World Health Organization.

**Supplementary Figure S1. The trend of late HIV diagnosis proportion and number of HIV cases between 2002 and 2024 in Yinzhou District, Ningbo.**

**Supplementary Figure S2. Kaplan-Meier survival curves comparing late HIV diagnosis and non-late HIV diagnosis groups in 120 months follow-up, numbers at risk were listed at each timepoint.**
